# Ultrasound-guided inferior alveolar nerve block for trismus during dental treatment: a case report

**DOI:** 10.1186/s40981-020-00400-0

**Published:** 2020-12-02

**Authors:** Yuki Kojima, Ryozo Sendo, Sachi Ohno, Mitsutaka Sugimura

**Affiliations:** Department of Dental Anesthesiology, Field of Oral and Maxillofacial Rehabilitation, Kagoshima University Graduate School of Medical and Dental Sciences, 8-35-1 Sakuragaoka, Kagoshima, 890-8544 Japan

**Keywords:** Jaw pain, Temporomandibular disorder, Trismus, Ultrasound-guided inferior alveolar nerve block

## Abstract

**Background:**

Temporomandibular disorder (TMD) is a broad term that encompasses pain and/or dysfunction of the masticatory musculature and TM joints (TMJs). When TMD becomes a chronic condition, the symptoms are extremely difficult to manage and require multiple interventions.

**Case presentation:**

A woman in her 50s developed TMD after a traffic accident 30 years ago. The patient presented with severe trismus due to TMJ pain and a maximum mouth opening of 20 mm. Ultrasound-guided inferior alveolar nerve block (IANB) was performed with ropivacaine. After IANB, the pain during mouth opening subsided and the maximum mouth opening improved to 40 mm. Dental treatment could be performed without difficulty and the patient could keep her mouth open throughout the treatment.

**Conclusions:**

Treatments for chronic TMD are limited and it is necessary to identify the precise etiology before choosing a treatment option. In this patient, ultrasound-guided IANB proved to be effective in relieving TMD-related trismus.

## Background

Temporomandibular disorder (TMD) is a broad term that encompasses pain and/or dysfunction of the masticatory musculature and temporomandibular joints (TMJs) [1]. The most important feature of TMD is pain followed by limited jaw movement and joint sounds [1]. Although TMD is not life threatening, its symptoms can greatly affect a person’s quality of life (QOL). When it becomes a chronic condition, the symptoms are extremely difficult to manage and often require multiple interventions.

The TMJ connects the mandible with the temporal bone [2]. The joint functions with the aid of the muscles and ligaments attached to the joint capsule, condylar neck, and mandibular body [3]. The TMJ is innervated by the trigeminal nerve, in which the mandibular branch (V3) provides motor nerve supply to the masticatory muscles while the auriculotemporal and masseteric branches provide sensory innervation to the joint [4]. Common symptoms of TMD are pain in the face, jaw, neck, and shoulders along with restricted jaw movements, headache, difficulty eating, bruxism, clenching, otalgia, and joint sounds [2]. Restricted jaw movement causes trismus, which leads to difficulty in eating and receiving dental treatments. Therefore, treatment of TMDs associated with trismus can significantly improve the QOL [1, 4].

Herein, we present the case report of a patient who developed TMD following a traffic accident and discuss the utility of ultrasound-guided inferior alveolar nerve block (IANB) in relieving trismus secondary to severe TMD.

## Case presentation

Written informed consent was obtained from the patient for publication of this case report and accompanying images.

A woman in her 50s presented with a medical history of generalized loss of muscle strength induced by post-traumatic stress disorder. At 27 years of age, she was involved in a traffic accident that resulted in cervical and lumbar disk herniation. Although spinal surgery was performed, the weakness in the left half of her body did not improve. Furthermore, it gradually progressed to loss of muscle strength and uterine and bladder prolapse; subsequently, she was diagnosed with disuse syndrome.

At the age of 39 years, she was diagnosed with diabetes and prescribed oral medications (metformin, Riobel); her HbA1c level at admission was 6.6%. She began developing asthma at 47 years of age and reported mild attacks approximately twice a month. Her regular therapy included theophylline and budesonide/formoterol inhalation. During an attack, she used a procaterol inhaler; however, she was prescribed oral steroids when the inhalers failed to control the symptoms. The patient developed ventricular extrasystoles at 53 years of age and was treated with oral disopyramide. She also had a history of anaphylactic shock, loss of consciousness, and respiratory arrest following administration of amoxicillin. She commented that, after losing consciousness in the hospital, she was monitored and given adrenaline. Since then, she has been prescribed an EpiPen and has been instructed to carry it with her.

The medical records from the examination conducted by the orthopedist 6 years ago indicated a Jackson test that showed radiating pain in the right upper limb. The left upper and lower limbs were found to be spastic owing to cervical myelopathy. MRI findings showed misalignment of the cervical spine. Stenosis of the spinal canal was observed owing to bulging of the intervertebral disk or osteophytes. Outer hernias were found on the left lateral side of C6/7 and on the right lateral side of C5/6.

The patient was scheduled to undergo extraction of a right lower molar and three upper anterior teeth secondary to progressing dental caries. However, it was anticipated that the treatment would be difficult since she could not open her mouth adequately. Therefore, we considered relieving the trismus to facilitate the dental treatment. Diagnostic imaging revealed no abnormality of TMJs. Palpation of the temporalis and masseter muscles revealed tenderness; therefore, the cause of the TMD was considered pain in the masticatory muscles during mouth opening. Consequently, it was expected that the mouth opening would improve if the pain were relieved.

**Fig. 1:**
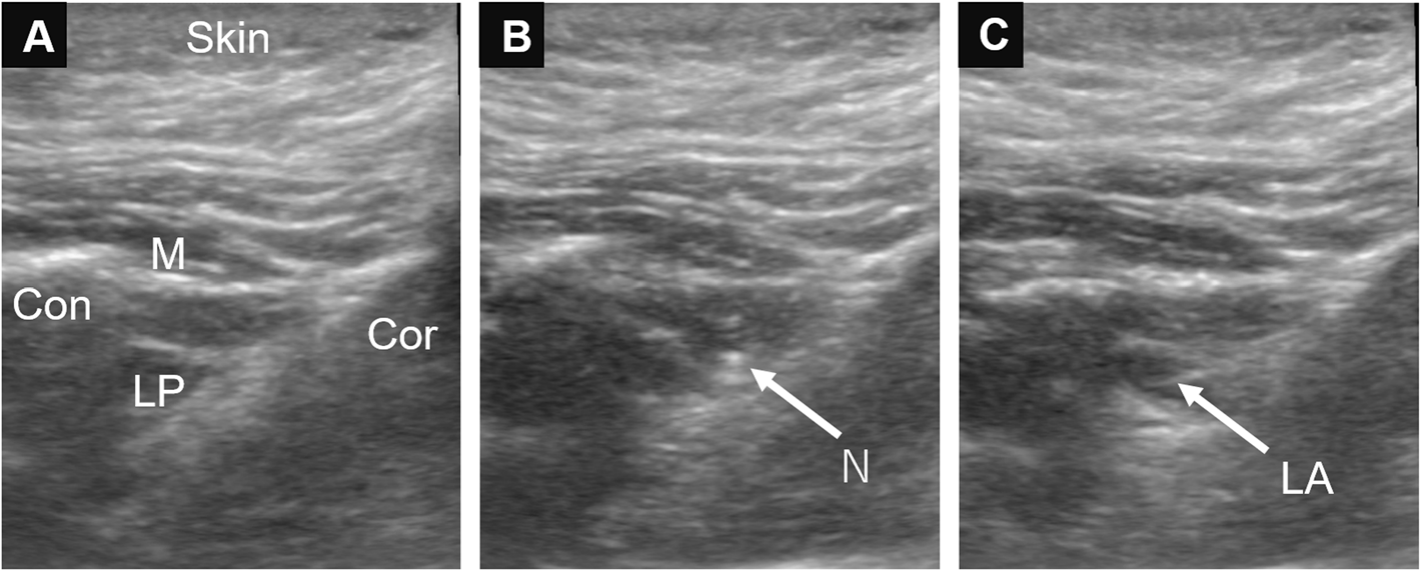
Ultrasound images and technique of inferior alveolar nerve block. A 22-G block needle was inserted using the lateral extraoral technique to infiltrate the inferior alveolar nerve. **a**, before needle insertion; Cor, coronoid; Con, condyle; M, masseter; LP, lateral pterygoid muscle; **b**, needle insertion; N, needle; **c**, after infiltration of local anesthesia; LA, local anesthesia

The maximum mouth opening was 20 mm between the upper alveolar crest and the lower anterior incisor apex before treatment. Mouth opening resulted in severe pain (visual analog scale pain score, 100/100) and was difficult to maintain. The patient was seated in the dental chair and monitoring of her blood pressure, oxygen saturation, and electrocardiogram was started. After confirming that her vital signs were normal, ultrasound-guided IANB was performed bilaterally with 6 ml of 0.375% ropivacaine on each side (Fig. 1). Three minutes later, the pain during mouth opening disappeared and mouth opening improved to 40 mm with a visual analog scale pain score of 10–11/100. Dental treatment could be performed without difficulty and the patient was able to maintain an open mouth throughout the treatment. The course of treatment and the patient’s recovery were unremarkable. No palsy or hypoesthesia was noted after the block. The patient informed us subsequently that the improvement in trismus lasted for 3 days following IANB.

## Discussion

According to the American Association of Dental Research, it is strongly recommended that unless there are specific and justifiable indications to the contrary, treatment of TMD should be based on the use of conservative, reversible, and evidence-based treatment modalities [2]. Treatment methods include gentle muscle massage, anti-inflammatory medications, and use of oral appliances [5, 6].

This patient developed TMD secondary to injury and had what can be considered a typical example of severe TMD [4]. Treatment should have been performed sooner since it became difficult due to the disease progression. Ultrasound-guided IANB was able to improve the trismus caused by TMD in this case.

In a recent study, Kumita et al. suggested that ultrasound-guided IANB was highly effective in perioperative analgesia in cases of gnathoplasty [7]. Another recent study found that ultrasound-guided IANB was useful in the perioperative management of patients undergoing mandibular sequestrectomy for medication-related osteonecrosis of the jaw [8]. Our results demonstrated that IANB provided effective pain control for 72 h. Furthermore, IANB was not associated with adverse events or prolonged hospitalization. These results also suggest that IANB provides effective postoperative analgesia following mandibular surgery. In the present case, the improvement in mouth opening lasted for 3 days after the procedure, which is consistent with previous reports; however, it is unclear as to why the analgesic effect persisted longer than the duration of action of the local anesthetic. The patient’s satisfaction with IANB was very high as it led to improved feeding. On the third day after the operation, the maximum mouth opening returned to that before IANB. Therefore, we are currently performing treatments such as opening training and manipulation after IANB. She is currently undergoing 1 month of treatment and mouth opening has improved to 45 mm.

Previous reports have described that a mandibular nerve block using the landmark technique was effective in relieving trismus for a short time in several patients [9–12]. These case reports indicated that the nerve block was effective for pain due to TMD; however, the classical approach carries a risk of vascular lesions or foramen ovale insertion. These complications can result in “total spinal anesthesia” and even pharyngeal penetration [9]. Ultrasound-guided IANB is much easier to perform, has no adverse effects, and may be useful for diagnostic purposes as well. This implies that the inability of a patient to open his/her mouth even after IANB points toward a disorder of the joint disk or deformation of the joint itself. The joint trigger block can be applied to the joint cavity. However, when the muscles involved in the TMJ result in as much pain as in this case, the effect of the joint trigger block may be limited. Since IANB is effective in the mandibular nerve innervation area, the range of effect is broad. The mandibular nerve innervates the masseter, temporalis, internal pterygoid, lateral pterygoid, mylohyoid, geniohyoid, digastric, and tensor veli palatini muscles, and its innervation area includes the mandibular teeth, lower jaw, lower lip, cheek, and tongue.

Further studies on the epidemiology, drug dosage of anesthetics, and accurate assessments of the duration of their action are required to guide the clinical decision regarding treatment of patients with trismus due to TMD.

## Data Availability

None.

## Abbreviations

IANB: Inferior alveolar nerve block
QOL: Quality of life
TMD: Temporomandibular disorder
TMJ: Temporomandibular joint
